# Case Report: A cluster of cases with rash associated with *Chlamydia pneumoniae* infection

**DOI:** 10.3389/fmed.2026.1922611

**Published:** 2026-09-03

**Authors:** Tianyi Wu, Gang Wang, Nannan Xu, Ke Xu, Gonghai Chen

**Affiliations:** 1 Department of Infectious Disease, Qilu Hospital, Cheeloo College of Medicine, Shandong University, Jinan, Shandong, China; 2 Shandong Key Laboratory of Medicine and Prevention Integration in Rheumatism and Immunity Disease, Jinan, Shandong, China

**Keywords:** *Chlamydia pneumoniae* infection, cluster outbreak, cutaneous manifestations, extrapulmonary manifestations, metagenomic next-generation sequencing

## Abstract

We report a case of clustered rashes among teenagers associated by *Chlamydia pneumoniae* (CP) infection. Most patients only presented with rashes without significant fever or other respiratory tract symptoms. Metagenomic next-generation sequencing (mNGS) of blood samples identified the culprit - the nucleic acid sequence of CP was detected in the peripheral blood of the index case. The results of targeted next-generation sequencing (t-NGS) of throat swabs further supported the presence of CP. After antibiotic treatment, the patients’ conditions improved. In this case, we should have a systematic understanding of rashes and mucositis caused by respiratory tract infections to facilitate further diagnosis and treatment.

## 1 Introduction

*Chlamydia pneumoniae* (CP) infection is relatively common in school-age children and accounts for approximately 10% of community-acquired pneumonia (CAP) cases (1). CP frequently involves both the upper and lower respiratory tracts and may cause pharyngitis, laryngitis, tonsillitis, sinusitis, bronchitis, and pneumonia. Pneumonia caused by CP is generally mild and is characterized by an insidious onset, no significant sex predilection, and year-round occurrence (2).

Although CP infection primarily affects the respiratory tract, a variety of extrapulmonary manifestations have been reported, including arthritis, myocarditis, pericarditis, encephalitis, Guillain–Barré syndrome, and dermatologic disorders (1, 3–6). These complications are generally uncommon compared with respiratory disease. Cutaneous manifestations appear to be rare and have been described mainly in isolated case reports and small case series. In the present study, we describe a rare cluster of rash cases among teenagers associated with CP infection. The patients initially presented with confluent erythematous rashes involving the face, neck, and forearms, with or without low-grade fever but without prominent respiratory symptoms. Physical examination revealed non-pruritic and non-vesicular cutaneous lesions, while microbiological investigations, including metagenomic next-generation sequencing (mNGS) and targeted next-generation sequencing (t-NGS), identified CP as the causative pathogen. This report expands the recognized spectrum of atypical CP manifestations and highlights the importance of considering CP infection in patients presenting predominantly with unexplained rash outbreaks.

## 2 Article types

Case report.

## 3 Case description

**Figure 1:**
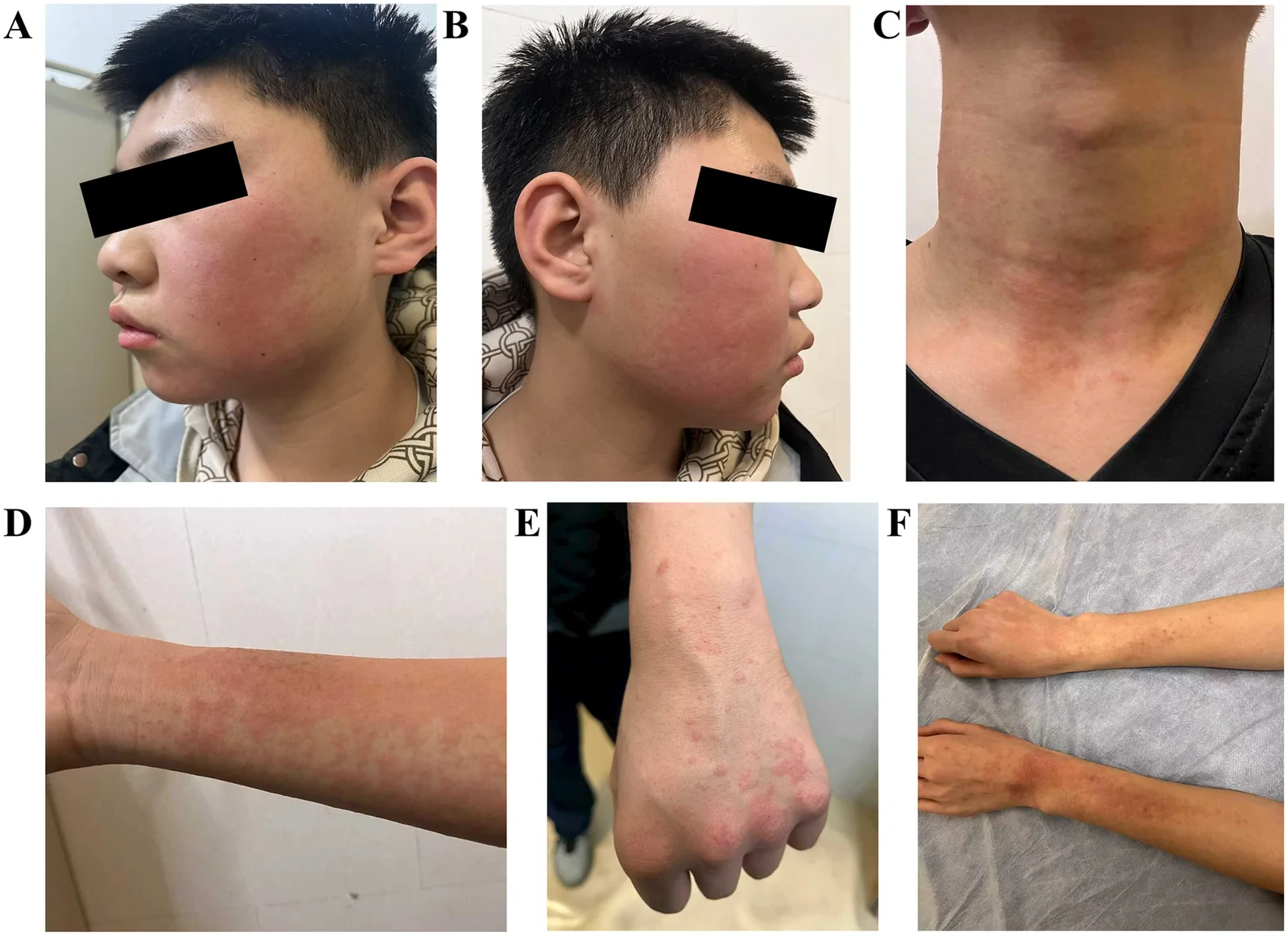
Clinical manifestations of cutaneous eruptions associated with *Chlamydia pneumoniae* infection. Confluent erythematous patches were observed on the face **(A,B)**, neck **(C)**, and bilateral forearms **(D–F)**. The lesions were diffuse, non-vesicular erythematous patches without obvious desquamation or ulceration.

In January 2025, a cluster of rash cases was identified among 30 teenage students from Shanxi Province who traveled to Zibo, Shandong Province, to participate in a volleyball competition. The index case developed symptoms on January 13, presenting with confluent erythematous patches predominantly involving the face, neck, and bilateral forearms, accompanied by fever (maximum axillary temperature, 38.3 °C) (Figure 1). The rash was non-pruritic and non-vesicular, with no accompanying respiratory, ocular, or gastrointestinal symptoms. The patient was treated with oral azithromycin (500 mg once daily) and antipyretic therapy; fever resolved, whereas the rash persisted.

Within four days of symptom onset in the index case (January 13–16, 2025), 13 additional patients developed similar cutaneous eruptions, with or without low-grade fever. Notably, none of the affected students reported significant respiratory symptoms during the course of illness. All affected individuals had close contact during training and shared accommodations. The overall attack rate was 47% (14/30).

An epidemiological investigation was initiated by the local Centers for Disease Control and Prevention (CDC) on January 16. Throat swabs and peripheral blood samples were collected for microbiological analysis. On January 17, several patients were referred to the Department of Infectious Diseases at Qilu Hospital. Metagenomic next-generation sequencing (mNGS) was performed on peripheral blood samples from the index case and the most recently affected case. Briefly, nucleic acids were extracted from peripheral blood specimens, followed by library preparation and high-throughput sequencing. Raw sequencing data underwent quality control, removal of low-quality reads, and subtraction of human host sequences through alignment to the human reference genome. The remaining non-human reads were aligned against comprehensive microbial reference databases for taxonomic classification and pathogen identification. Detection of pathogen-specific sequence reads significantly above background levels and consistent with the clinical presentation was considered supportive evidence of infection. To investigate potential alternative infectious etiologies, all affected patients underwent multiplex respiratory pathogen testing, serological testing for *Mycoplasma pneumoniae* (MP) and CP, and screening for cytomegalovirus (CMV) and Epstein–Barr virus (EBV). Blood cultures were also performed in patients presenting with fever. All results were negative.

On January 19, mNGS detected nucleic acid sequences of CP in the peripheral blood of the index case. Serological testing for CP-specific IgM and IgG antibodies was initially negative, likely due to early sampling prior to seroconversion. To validate these findings, throat swab samples were submitted to KingMed Diagnostics. The t-NGS assay was designed to detect *C. pneumoniae*-specific genomic sequences in respiratory specimens and confirmed the presence of CP nucleic acid in the tested samples, providing additional microbiological evidence supporting CP infection (Supplementary Table S1).

Although molecular confirmation of CP infection was obtained only in a subset of patients, including the index case and recently identified incident cases, the remaining patients were classified as epidemiologically linked cases based on compatible clinical manifestations and shared exposure history during the outbreak. All patients received azithromycin-based therapy and supportive care. The rashes resolved within 5–7 days, and all patients recovered without complications. Follow-up serum samples obtained 30 days after symptom onset from the index case and the most recently affected case demonstrated seroconversion by enzyme-linked immunosorbent assay (ELISA).

The outbreak was effectively contained following CDC intervention, including isolation of symptomatic individuals, environmental disinfection, and health education. No secondary transmission was identified.

## 4 Discussion

Cutaneous rashes associated with CP infection are a rare extrapulmonary manifestation, described predominantly in isolated case reports and small case series. The underlying mechanism is believed to involve immune-mediated reactions rather than direct microbial invasion (7). CP infection typically has an insidious onset; consistent with this, most of the students in the present cluster exhibited only cutaneous lesions without significant fever or other systemic symptoms. In the early phase, CP infection generally presents as a mild upper respiratory tract illness resembling *M. pneumoniae* infection and may occasionally be accompanied by extrapulmonary complications, including arthritis, myocarditis, pericarditis, encephalitis, and Guillain–Barré syndrome (1, 3–6).

Interestingly, cutaneous eruptions are also recognized extrapulmonary features of MP. Historically, MP-associated mucocutaneous eruptions were frequently classified as Stevens–Johnson syndrome (SJS), toxic epidermal necrolysis (TEN), or erythema multiforme (EM); however, these classifications did not adequately reflect the distinct clinical features and generally favorable prognosis of MP-associated disease. Canavan et al. proposed the term *Mycoplasma pneumoniae*-induced rash and mucositis (MIRM) to define this unique clinical entity, characterized primarily by prominent mucosal involvement—most commonly affecting the oral, ocular, and genitourinary mucosa—and with relatively limited skin manifestations (8). In 2019, the Pediatric Dermatology Research Alliance introduced the broader term reactive infectious mucocutaneous eruption (RIME) to encompass MIRM and other clinically similar para-infectious mucosal-predominant eruptions (9). This expanded framework has improved understanding of immune-mediated mucocutaneous diseases secondary to respiratory pathogens. Recent evidence further supports the concept that MIRM represents an immune-mediated reaction triggered by respiratory pathogens rather than direct microbial invasion of epithelial tissues. Proposed mechanisms include pathogen-induced immune activation, cytokine-mediated inflammation, and subsequent mucosal tissue injury (10). A recent systematic review and case series including patients with confirmed MP-associated mucositis highlighted that oral involvement is the dominant clinical feature of MIRM, with severe ulcerations, hemorrhagic crusting, and functional impairment frequently reported. These findings reinforce the importance of recognizing respiratory infection–associated immune-mediated mucocutaneous diseases as a distinct clinical spectrum (10).

Although the patients in our cluster did not exhibit mucosal involvement and therefore do not fulfill the clinical characteristics of MIRM, the MIRM concept provides an important framework for understanding how respiratory pathogens may induce extrapulmonary immune responses. Similar to MP, CP is capable of causing extrapulmonary manifestations, and the present cluster suggests that CP infection may also trigger a predominantly cutaneous immune-mediated response. Further studies are needed to clarify whether CP-associated skin eruptions represent a related but distinct phenotype within the broader spectrum of respiratory infection–associated immune-mediated dermatological manifestations.

The differential diagnosis of clustered rashes among adolescents includes infectious exanthems, allergic or drug-related eruptions, and environmental or contact-related dermatitis. In this cluster, several clinical, epidemiological, and laboratory findings helped narrow the differential diagnosis. The absence of typical prodromal symptoms, mucosal involvement, and characteristic rash patterns reduced the likelihood of common viral exanthems such as measles, rubella, varicella, and enterovirus-associated eruptions. Furthermore, no history of newly introduced medications, dietary changes, or identifiable shared environmental exposures was reported, arguing against drug eruptions, allergic reactions, and contact dermatitis. Alternative infectious causes were also systematically evaluated. Multiplex respiratory pathogen testing, screening for cytomegalovirus (CMV) and Epstein–Barr virus (EBV), and blood cultures obtained from febrile patients were all negative. These findings helped exclude several common viral, bacterial, and atypical infectious etiologies. Given the persistent epidemiological clustering and the absence of an alternative diagnosis, further molecular investigations were undertaken. Subsequently, CP nucleic acid was detected by both metagenomic mNGS of peripheral blood and t-NGS of throat swab specimens. Follow-up serological testing demonstrated seroconversion in the investigated patients, providing additional evidence supporting recent CP infection. Taken together, the epidemiological, molecular, and serological findings support CP as the most likely pathogen associated with this outbreak. Nevertheless, because molecular confirmation was not obtained in all patients and the sample size was limited, the contribution of other unidentified factors cannot be completely excluded. Diagnostic approaches for CP include serology, cell culture, and nucleic acid–based molecular testing. Serological assays detecting CP-specific IgM and IgG antibodies remain widely used because of their low cost; however, their diagnostic utility is limited in early infection due to delayed antibody production and suboptimal specificity when used alone. Confirmation typically requires paired sera from the acute and convalescent phases. In contrast, molecular methods demonstrate higher sensitivity and specificity and are increasingly recommended in clinical practice (11, 12). In cases with negative serology but high clinical suspicion, cell culture or metagenomic next-generation sequencing (mNGS) may be employed to reduce the risk of false-negative results. In the present outbreak, serological testing was negative, likely due to early sampling prior to seroconversion, whereas next-generation sequencing provided critical evidence supporting CP infection. Given that serological assays may yield false-negative results within the first three weeks after infection, polymerase chain reaction (PCR)–based detection of CP DNA from respiratory specimens is also recommended as a sensitive and specific diagnostic approach (13).

This cluster highlights the potential for CP infection to induce immune-mediated cutaneous manifestations and underscores the diagnostic value of next-generation sequencing in identifying atypical or early-stage infections when conventional serological methods are inconclusive.

## 5 Limitations

Although contamination and false-positive signals are recognized limitations of sequencing-based diagnostic methods, the likelihood of a spurious result in the present study is reduced by several factors. The mNGS assay was performed as a clinically validated diagnostic test, and the detected number of CP sequence reads exceeded the laboratory reporting threshold for positivity. Furthermore, the findings were corroborated by targeted next-generation sequencing of throat swab specimens and by subsequent seroconversion in follow-up serum samples. Nevertheless, molecular confirmation was not available for all affected patients, and the small sample size limits the strength of the observed association between CP infection and the rash outbreak.

## 6 Conclusion

The clustered rash outbreak among teenagers was associated with CP infection. Given that CP is primarily transmitted via respiratory droplets, close contact among students during the competition likely facilitated person-to-person transmission. Similar to *M. pneumoniae*, CP infection can occasionally present with immune-mediated cutaneous manifestations in addition to respiratory involvement.

This outbreak underscores the importance of timely pathogen identification, coordinated collaboration between medical institutions and public health authorities (e.g., Centers for Disease Control and Prevention), and the implementation of rapid, evidence-based interventions to effectively contain emerging infectious clusters.

## Data Availability

The original contributions presented in the study are included in the article/Supplementary Material, further inquiries can be directed to the corresponding author.
