# Supplementary material for: Case Report: A cluster of cases with rash associated with Chlamydia pneumoniae infection
Source: Front Med (Lausanne). 2026 Sep 3;13:1922611. doi: 10.3389/fmed.2026.1922611 (PMC13581956; doi:10.3389/fmed.2026.1922611)
Supplement: Supplementary file 1 [file Table1.docx]

# Supplementary Table S1. Clinical characteristics and laboratory findings of the 14 affected patients

| Patient ID | Age/Sex | Symptom onset | Clinical manifestations | Rash distribution | Rash duration (days) | CP serology | mNGS (blood) | t-NGS (throat swab) |
| --- | --- | --- | --- | --- | --- | --- | --- | --- |
| P1 | 15/M | Jan 13, 2025 | Rash, fever (38.3°C) | Face, neck, bilateral forearms | 13 | IgM+/IgG+ (Day 30) | CP detected (10 reads) | Positive |
| P2 | 14/M | Jan 14, 2025 | Rash only | Neck, bilateral forearms | 13 | ND | ND | ND |
| P3 | 15/M | Jan 14, 2025 | Rash, fever (37.9°C) | Neck, bilateral dorsal hands and forearms | 14 | ND | ND | ND |
| P4 | 15/F | Jan 14, 2025 | Rash, fever (37.5°C) | Bilateral forearms | 12 | ND | ND | ND |
| P5 | 15/M | Jan 14, 2025 | Rash only | Face, bilateral forearms | 12 | ND | ND | ND |
| P6 | 14/M | Jan 14, 2025 | Rash, fever (37.6°C) | Bilateral dorsal hands and forearms | 12 | ND | ND | ND |
| P7 | 14/M | Jan 14, 2025 | Rash only | Bilateral forearms | 13 | ND | ND | ND |
| P8 | 15/M | Jan 15, 2025 | Rash only | Bilateral forearms | 10 | ND | ND | ND |
| P9 | 15/F | Jan 15, 2025 | Rash, fever (37.3°C) | Bilateral dorsal hands and forearms | 12 | ND | ND | ND |
| P10 | 15/M | Jan 15, 2025 | Rash only | Bilateral forearms | 10 | ND | ND | ND |
| P11 | 15/M | Jan 15, 2025 | Rash, fever (37.5°C) | Face, bilateral dorsal hands and forearms | 12 | IgM+/IgG+ (Day 30) | Not detected | Weakly positive |
| P12 | 15/M | Jan 15, 2025 | Rash only | Bilateral forearms | 12 | ND | ND | ND |
| P13 | 13/M | Jan 16, 2025 | Rash, fever (37.8°C) | Face, neck, bilateral forearms | 12 | IgM+/IgG+ (Day 30) | Not detected | Not detected |
| P14 | 15/M | Jan 16, 2025 | Rash only | Bilateral forearms | 11 | ND | ND | ND |

Abbreviations: CP, Chlamydia pneumoniae; mNGS, metagenomic next-generation sequencing; t-NGS, targeted next-generation sequencing; ND, not determined.
